# Low-Dose *Aronia melanocarpa* Concentrate Attenuates Paraquat-Induced Neurotoxicity

**DOI:** 10.1155/2016/5296271

**Published:** 2015-12-06

**Authors:** A. J. Case, D. Agraz, I. M. Ahmad, M. C. Zimmerman

**Affiliations:** ^1^Department of Cellular and Integrative Physiology, University of Nebraska Medical Center, Omaha, NE 68198-5850, USA; ^2^College of Allied Health Professions, University of Nebraska Medical Center, Omaha, NE 68198, USA; ^3^Redox Biology Center, University of Nebraska Lincoln, Lincoln, NE 68588, USA

## Abstract

Herbicides containing paraquat may contribute to the pathogenesis of neurodegenerative disorders such as Parkinson's disease. Paraquat induces reactive oxygen species-mediated apoptosis in neurons, which is a primary mechanism behind its toxicity. We sought to test the effectiveness of a commercially available polyphenol-rich* Aronia melanocarpa* (aronia berry) concentrate in the amelioration of paraquat-induced neurotoxicity. Considering the abundance of antioxidants in aronia berries, we hypothesized that aronia berry concentrate attenuates the paraquat-induced increase in reactive oxygen species and protects against paraquat-mediated neuronal cell death. Using a neuronal cell culture model, we observed that low doses of aronia berry concentrate protected against paraquat-mediated neurotoxicity. Additionally, low doses of the concentrate attenuated the paraquat-induced increase in superoxide, hydrogen peroxide, and oxidized glutathione levels. Interestingly, high doses of aronia berry concentrate increased neuronal superoxide levels independent of paraquat, while at the same time decreasing hydrogen peroxide. Moreover, high-dose aronia berry concentrate potentiated paraquat-induced superoxide production and neuronal cell death. In summary, aronia berry concentrate at low doses restores the homeostatic redox environment of neurons treated with paraquat, while high doses exacerbate the imbalance leading to further cell death. Our findings support that moderate levels of aronia berry concentrate may prevent reactive oxygen species-mediated neurotoxicity.

## 1. Introduction

Neurodegeneration is a hallmark of numerous neurological disorders such as age-related dementia, Alzheimer's disease, and Parkinson's disease [1]. While several etiologies have been identified leading to the loss of neurons, one possible contributing factor is contact with environmental toxins [2]. A major source of these poisons in rural farming areas is insecticides and herbicides, and exposure to these has been suggested as a major risk factor for neurological diseases such as Parkinson's disease [3, 4]. One commonly used compound in herbicides is paraquat (PQ), and extensive research has demonstrated a direct link between neurotoxicity and PQ contact [5–7]. PQ is a known redox cycling agent that impacts complex I activity of the mitochondria, increases superoxide (O_2_^•−^) production, and decreases endogenous antioxidant capacity leading to increased neurotoxicity through apoptosis [8, 9]. Several studies have examined the effects of single antioxidant supplementation in the amelioration of PQ-induced neurotoxicity [10–12], but to date it remains unclear how combinations of small molecule antioxidants gained through dietary or nutritional means affect this toxin-mediated neuron loss.

*Aronia melanocarpa*, also known as black chokeberries or simply aronia berries, are small, dark, cherry-like berries belonging to the plant family Rosaceae [13]. Aronia berries are native to Eastern Europe and the Eastern United States but have recently become cultivated in large quantities by Midwest farmers. The berries have garnered much attention by the general public due to their significantly high quantity of polyphenols, in particular anthocyanins and flavonoids, which are estimated at 2-3 times greater amounts than in comparable berries [14, 15]. Polyphenols, such as resveratrol and quercetin, have been shown to possess significant antioxidant properties by both directly scavenging reactive oxygen species (ROS) and inducing cellular antioxidant systems to help combat oxidative environments [15]. Aronia berries are no exception, and a widespread literature exists examining the potential beneficial effect of aronia berries on diseases including hypercholesterolemia, cancer, diabetes, and inflammation [16–19]. However, the vast majority of these studies only examine enriched extracts of the polyphenols from aronia berries and not the effects of the whole berry or berry concentrate in the disease models. Moreover, a dearth of studies exists examining the potential beneficial effects of aronia berries on diseases affecting the nervous system.

Herein, we tested the hypothesis that polyphenolic-rich aronia berry concentrate (AB) has antioxidant protective effects against ROS-induced neurotoxicity by PQ. Utilizing a neuronal cell culture model, we indeed demonstrate AB protects against PQ-induced cellular toxicity and an increase in ROS. However, we show that only low doses of AB demonstrate this protective effect, while high doses potentiate the negative effects elicited by PQ. Overall, this work suggests a proper balance of prooxidants and antioxidants are required for normal neuronal homeostasis, and moderate levels of AB shift the balance in favor of neuronal survival following PQ exposure.

## 2. Materials and Methods

### 2.1. Cell Culture and Reagents

NG108-15 neuroblastoma cells (ATCC #HB-12317) were cultured and maintained in RPMI 1640 (Gibco #11875-093, Grand Island, NY) supplemented with 10% fetal bovine serum (Atlanta Biologicals #S11150, Lawrenceville, GA) and 1% penicillin/streptomycin (Gibco #15140-122, Grand Island, NY). As per manufacturer's instructions for human consumption, the aronia berry concentrate (Superberries/Mae's Health and Wellness-Westin Foods, Omaha, NE) was diluted to the drinking concentration (1 : 16 in culture media) prior to making serial working dilutions. Paraquat (Sigma-Aldrich #36541, St. Louis, MO) was diluted in double-distilled water and filter-sterilized prior to use. Cells were plated (200,000 cells/60 mm dish) 24 hours prior to counting or treatment at 0 hours. For AB + PQ experiments, AB was started at 0 hours and PQ was started at 24 hours; pretreatment was performed to examine the protective effects of AB to PQ toxicity. Media were made fresh and changed daily.

### 2.2. Growth Curves and Apoptosis Assays

For growth curve analyses, cells were washed twice to remove unattached dead cells. Remaining live and attached cells were scrape harvested, isolated by centrifugation, and counted using size exclusion on a Beckman Coulter counter [20]. Apoptotic fraction of live cells was performed on the same cell population using the Alexa Fluor 488 annexin V/Dead Cell Apoptosis Kit (Molecular Probes #V13241, Grand Island, NY) as per manufacturer's instructions [21]. Briefly, freshly isolated cells were incubated with an Alexa Fluor 488-conjugated annexin V antibody as well as propidium iodide (PI). Cells were analyzed on a FACSCalibur flow cytometer (Becton Dickinson, Franklin Lakes, NJ) at 488 nm excitation and 535 and 610 emission for annexin V and PI, respectively. Apoptotic fraction was considered as cells that were annexin V positive, while remaining are PI negative.

### 2.3. Superoxide Quantification

Cells were resuspended in serum-free, phenol red-free RPMI 1640 (Gibco #11835-030, Grand Island, NY) with 10 *μ*M dihydroethidium (DHE; VWR #101447-534, Chicago, IL) for measuring total cellular O_2_^•−^ levels or with 10 *μ*M MitoSOX (Life Technologies #M36008, Grand Island, NY) to measure mitochondrial-specific O_2_^•−^ levels and incubated for 30 min at 37°C. Following this, cells were immediately centrifuged at 4°C and resuspended in ice-cold serum-free, phenol red-free media. Cells were analyzed immediately on a LSRII Green Laser flow cytometer (Becton Dickinson, Franklin Lakes, NJ) at 488 nm excitation and 610 nm emission and quantified using FlowJo cytometric analysis software (Tree Star, Ashland, OR) [20].

### 2.4. Hydrogen Peroxide (H_2_O_2_) Quantification

Replication-deficient recombinant adenoviruses (Ad5-CMV) encoding either HyPer Cyto (Cytoplasm-targeted HyPer construct; Evrogen #FP941, Moscow, Russia) or HyPer-Mito (Mitochondria-targeted HyPer construct, Evrogen #FP942, Moscow, Russia) were purchased from the University of Iowa Viral Vector Core Facility (Iowa City, IA). After plating, cells were transduced with 100 multiplicity of infection (MOI; transduction efficiency measured at 95.4% ± 3.2% by flow cytometry with negligible toxicity) of respective virus for 24 hours in serum-free media prior to treatment with AB or PQ. Following treatment, cells were analyzed immediately on a LSRII Green Laser flow cytometer at 488 nm excitation and 509 nm emission and quantified using FlowJo cytometric analysis software [22].

### 2.5. Western Blotting Analysis

Immunoblotting was performed on whole cell lysates. Samples were separated on 10% denaturing gels, followed by a transfer to nitrocellulose membranes. After blocking in 5% milk in Tris-Buffered Saline and Tween 20, membranes were incubated with primary antibody (copper/zinc superoxide dismutase, CuZnSOD, 1 : 1000 dilution, Santa Cruz Biotechnology, Santa Cruz, CA; manganese superoxide dismutase, MnSOD, 1 : 1000 dilution, Upstate Biotech/Millipore, Billerica, MA; catalase, 1 : 1000 dilution, Abcam, Cambridge, MA; NADPH oxidase 2, Nox2, 1 : 500 dilution, Santa Cruz Biotechnology, Santa Cruz, CA; NADPH oxidase 4, Nox4, 1 : 500 dilution, Novus Biologicals, Littleton, CO; *β*-actin, 1 : 1000 dilution, Sigma-Aldrich, St. Louis, MO) overnight at 4°C. Following washout of primary antibody, membranes were incubated with secondary antibody (1 : 10,000, Thermo Scientific, Rockford, IL) for 1 hour at room temperature. After addition of chemiluminescence substrate (SuperSignal West Femto, Thermo Scientific, Rockford, IL), images were acquired on a UVP Bioimaging System (UVP LLC, Upland, CA) [23].

### 2.6. Antioxidant Activity Gels

Activity gels were run utilizing whole cell lysates. Samples were separated on 12% nondenaturing gels with ammonium persulfate used as the polymerization catalyst in the running gel and riboflavin-light in the stacking gel. Gels were prerun for one hour at 4°C prior to sample loading. For superoxide dismutase activity, the gel was stained in a solution containing 2.43 mM nitroblue tetrazolium, 28 mM tetramethylethylenediamine, and 25 *μ*M riboflavin-5′-phosphate for 20 minutes at room temperature protected from light. Following this incubation, the gel was rinsed thrice with double-distilled water and allowed to expose under fluorescent light. For catalase activity, the gel was first allowed to incubate in a 0.003% H_2_O_2_ solution for 10 minutes prior to staining with 2% ferric chloride and 2% potassium ferricyanide. Gel images were obtained by scanning using a Brother MFC-8870DW scanner [24].

### 2.7. Glutathione Assay

Oxidized (GSSG) as well as reduced (GSH) glutathione was measured using the GSSG/GSH Quantification kit (Dojindo Molecular Technologies #G257-10, Rockville, MD) as per manufacturer's instructions. Briefly, the assay is based on the glutathione dependent reduction of 5,5′-dithiobis-2-nitrobenzoic acid to 5-mercapto-2-nitrobenzoic acid (*λ*_max_: 415 nm). Absorbance was measured at 415 nm using a SpectraMax M5 multimode plate reader (Molecular Devices, Sunnyvale, CA) [25].

### 2.8. Statistics

Data are presented as mean ± standard error of the mean (SEM). For two group comparisons, Student's *t*-test was used. For multiple group comparisons, one-way ANOVA followed by Newman-Keuls posttest was used. GraphPad Prism 5.0 statistical and graphing software was used for all analyses. Differences were considered significant at *p* < 0.05.

## 3. Results

### 3.1. AB Protects Neurons from PQ-Induced Cell Death

**Figure 1:**
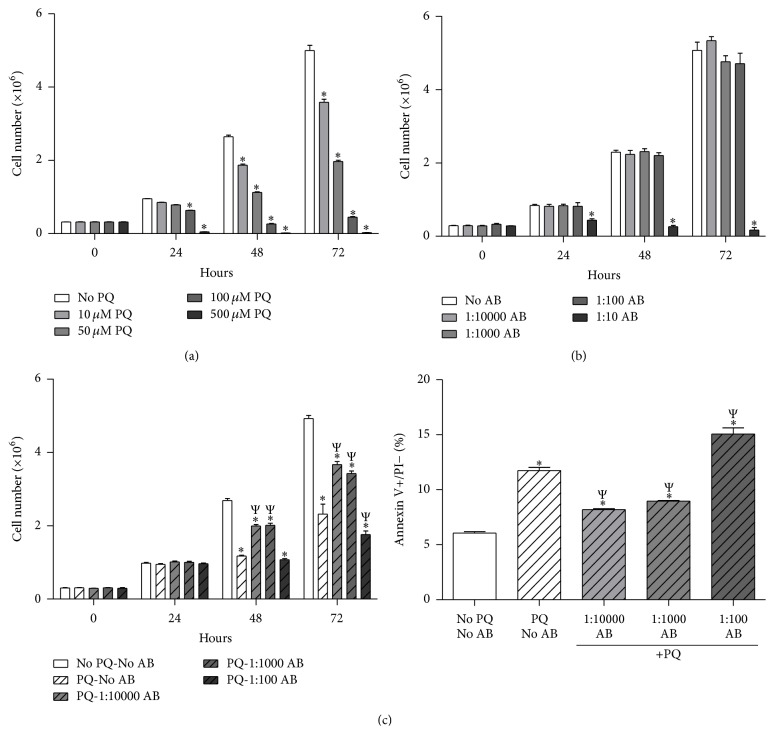
AB attenuates PQ-induced neurotoxicity. NG108-15 cells were treated with various doses of PQ, AB, or AB + PQ for 72 hours. (a) Growth curve of NG108-15 cells with increasing doses of PQ. IC_50_ of PQ calculated at approximately 50 *μ*M. *N* = 6. (b) Growth curve of NG108-15 cells with increasing doses of AB. 1 : 10 AB demonstrated significant toxicity and thus was not used in further studies. *N* = 6. (c) Left panel, growth curve of NG108-15 cells with 50 *μ*M of PQ along with various doses of AB. AB was added at 0 hours, while PQ was added at 24 hours after plating. *N* = 6. Right panel, analysis of apoptotic NG108-15 cells with 50 *μ*M of PQ along with various doses of AB. Apoptotic fraction was defined as annexin V positive and propidium iodide (PI) negative. *N* = 4. ^*∗*^ *p* < 0.05 versus No PQ or AB; ^Ψ^ *p* < 0.05 versus PQ-No AB.

PQ is a well-established neurotoxin known to induce neuron cell death by ROS-mediated apoptosis [26]. To identify an appropriate dose of PQ required to induce neurotoxicity in our neuronal cell culture model, we performed growth curves in the presence of increasing amounts of PQ and identified the IC_50_ of PQ to be approximately 50 *μ*M (Figure 1(a)). Additionally, to understand if AB alone had any effects on cellular viability we exposed cells to increasing concentrations of AB in 10-fold serial dilutions (Figure 1(b)). Only the highest dose tested (i.e., 1 : 10 AB) demonstrated significant toxicity to the cells and thus was not used in further studies. Last, to identify if AB had any effect on attenuating PQ-induced neurotoxicity, we treated cells with various dilutions of AB with 50 *μ*M PQ (Figure 1(c), left panel). Interestingly, only the lowest concentrations of AB (i.e., 1 : 1000 and 1 : 10000) demonstrated significant rescuing effects on the PQ-treated cells. In contrast, the highest concentration of AB (i.e., 1 : 100) potentiated the PQ-induced cell death at 72 hours. Furthermore, low doses of AB decreased, while high doses of AB exacerbated the apoptotic fraction of PQ-treated NG108-15 cells (Figure 1(c), right panel). Taken together, these data suggest that lower doses of AB have protective effects against PQ-induced neurotoxicity.

### 3.2. PQ-Induced Increase in O_2_^•−^_ _ Levels Is Attenuated by Low-Dose AB

**Figure 2:**
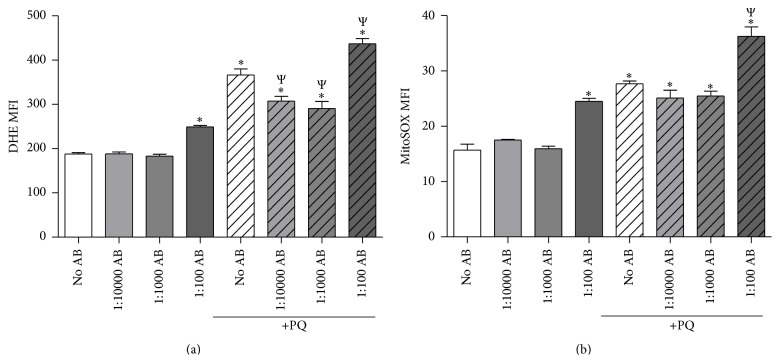
Low-dose AB decreases PQ-induced increase in O_2_^•−^ levels. NG108-15 cells were treated with 50 *μ*M PQ with various doses of AB. AB was added 24 hours prior to PQ; cells were analyzed 48 hours after PQ administration. (a) Total cellular O_2_^•−^ levels measured by dihydroethidium (DHE) oxidation and flow cytometry. *N* = 4. (b) Mitochondrial-specific O_2_^•−^ levels measured by MitoSOX oxidation and flow cytometry. MFI = mean fluorescence intensity. *N* = 4. ^*∗*^ *p* < 0.05 versus No AB; ^Ψ^ *p* < 0.05 versus PQ-No AB.

The primary and direct ROS generated by PQ is O_2_^•−^. We first measured total cellular O_2_^•−^ utilizing the O_2_^•−^-sensitive probe DHE (Figure 2(a)). As expected, PQ alone increased DHE oxidation roughly 2-fold. Interestingly, low-dose AB significantly attenuated the PQ-induced increase in O_2_^•−^ levels, while high-dose AB exacerbated this response. In addition, high-dose AB alone significantly increased DHE oxidation in the absence of PQ. Next, because PQ is known to play a role in the direct generation of mitochondrial-localized O_2_^•−^, we measured mitochondrial-specific O_2_^•−^ levels using MitoSOX Red (Figure 2(b)). Similar to what we observed with total cellular O_2_^•−^ levels, PQ alone also significantly increased mitochondrial O_2_^•−^ levels. Low-dose AB moderately decreased these levels, but these differences were not statistically significant. Additionally, high-dose AB alone increased mitochondrial O_2_^•−^ levels and once again intensified PQ-induced mitochondrial O_2_^•−^. In summary, these data suggest that low, but not high, doses of AB may have antioxidant effects that reduce the PQ-induced increase in neuronal O_2_^•−^ levels.

### 3.3. AB Alters Steady-State Cellular H_2_O_2_ Levels

**Figure 3:**
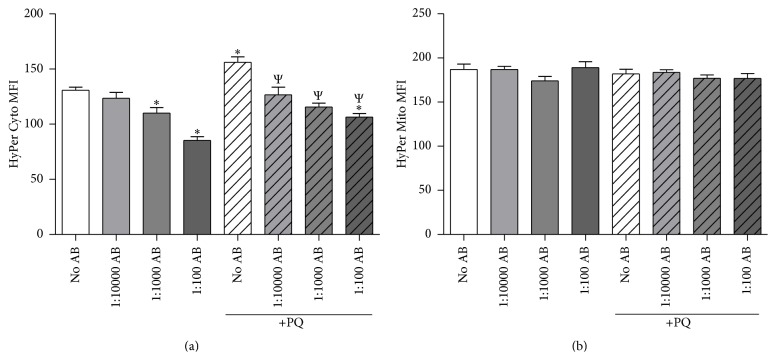
AB decreases cellular H_2_O_2_ in a dose-dependent fashion. NG108-15 cells were treated with 50 *μ*M PQ with various doses of AB. AB was added 24 hours prior to PQ; cells were analyzed 48 hours after PQ administration. (a) Cytoplasmic H_2_O_2_ levels measured by a cytoplasmic-targeted H_2_O_2_-sensitive fluorescent protein (HyPer Cyto). *N* = 4. (b) Mitochondrial H_2_O_2_ levels measured by a mitochondria-targeted H_2_O_2_-sensitive fluorescent protein (HyPer Mito). MFI = mean fluorescence intensity. *N* = 4. ^*∗*^ *p* < 0.05 versus No AB; ^Ψ^ *p* < 0.05 versus PQ-No AB.

O_2_^•−^ is a short lived species that is spontaneously and enzymatically (by superoxide dismutases) converted to H_2_O_2_ [27]. To assess intracellular H_2_O_2_ levels, we utilized fluorescent proteins that increase in fluorescence when oxidized specifically by H_2_O_2_ (i.e., HyPer) [22]. First, using a cytoplasm-targeted HyPer (HyPer Cyto) we observed a dose-dependent decrease in cytoplasmic H_2_O_2_ levels with increased concentration of AB alone (Figure 3(a)). PQ treatment led to a small but significant increase in cytoplasmic H_2_O_2_ levels, and this response was attenuated with increasing doses of AB. Neither PQ nor AB had any effect on mitochondrial-localized H_2_O_2_ levels as measured by the mitochondrial-targeted HyPer construct (Hyper Mito; Figure 3(b)). These data suggest that AB has potent H_2_O_2_ scavenging effects under both normal, nonoxidative stress and PQ-induced oxidative stress conditions.

### 3.4. AB Has a Minimal Effect on Prooxidant and Antioxidant Enzyme and Activity Levels

**Figure 4:**
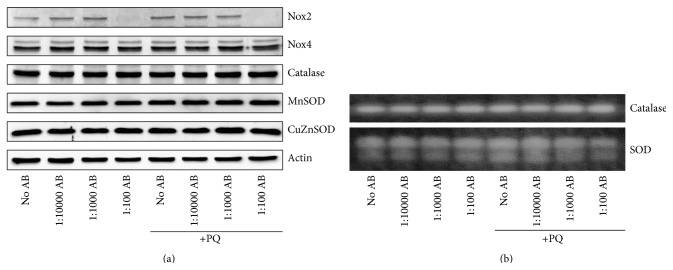
High-dose AB decreases Nox2 expression but does not affect other cellular antioxidant or prooxidant enzyme systems. NG108-15 cells were treated with 50 *μ*M PQ with various doses of AB. AB was added 24 hours prior to PQ; cells were analyzed 48 hours after PQ administration. (a) Western blot analysis of the major neuronal prooxidant enzymes NADPH oxidases 2 and 4 (Nox2 and Nox4) and antioxidant enzymes catalase, manganese superoxide dismutase (MnSOD), and copper/zinc superoxide dismutase (CuZnSOD). (b) In-gel activity assay for catalase and SOD demonstrating no change with any treatment course. Images are representative of 4 separate experiments; with the exception of Nox2, no significant changes were observed upon quantification.

The decrease in ROS observed by the addition of AB may be due to direct scavenging of ROS or by the alteration of endogenous antioxidant or prooxidant enzyme systems. First, we performed western blot analyses on whole cell lysates and observed no significant changes in the protein levels of cytoplasmic CuZnSOD, mitochondrial MnSOD, or the peroxisomal H_2_O_2_ removing enzyme catalase (Figure 4(a)). Because polyphenolic compounds like those found in AB have been shown to activate the sirtuin class of enzymes [28], which may alter the activity of endogenous antioxidant enzymes [29], we further examined antioxidant enzyme activities for both SOD and catalase and observed no significant differences in any treatment group (Figure 4(b)). In addition to exploring endogenous antioxidant systems, we also investigated the prooxidant NADPH oxidase (Nox) family of enzymes, which contribute to the production and steady-state levels of cellular O_2_^•−^ and H_2_O_2_ levels. Examining the catalytic subunits of the two major Nox enzymes found in neurons (i.e. Nox2 and Nox4) we observed a substantial reduction in the amount of immunoreactivity for Nox2 with high-dose AB independent of PQ treatment (Figure 4(a)), but no changes were observed with lower doses. Taken together, while high-dose AB appears to have an effect on Nox2 levels, overall, AB does not appear to have a significant impact on the endogenous antioxidant or prooxidant enzyme systems in our neuronal cell culture model.

### 3.5. PQ-Induced Oxidized Glutathione Is Significantly Reduced with Low-Dose AB

**Figure 5:**
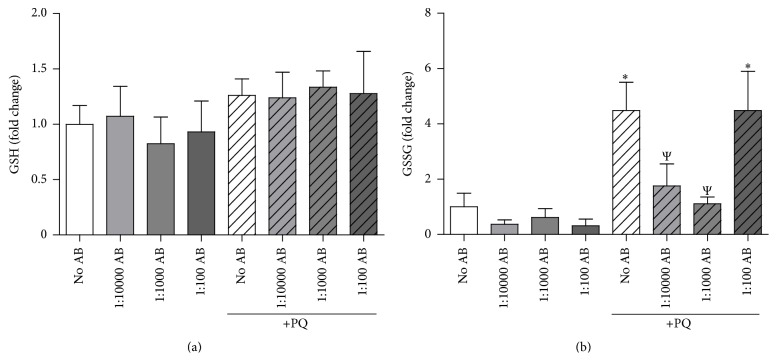
Low-dose AB rescues neurons from PQ-mediated increases in oxidized glutathione (GSSG). NG108-15 cells were treated with 50 *μ*M PQ with various doses of AB. AB was added 24 hours prior to PQ; cells were analyzed 48 hours after PQ administration. (a) Relative levels of reduced glutathione (GSH). *N* = 3. (b) Relative levels of oxidized glutathione (GSSG). *N* = 3. ^*∗*^ *p* < 0.05 versus No AB; ^Ψ^ *p* < 0.05 versus PQ-No AB.

In addition to antioxidant enzyme systems, the cell is home to numerous small molecule antioxidant systems. The most abundant small molecule antioxidant system in the cell is glutathione, which may be cycled between a reduced and oxidized state depending on the redox environment of the cell and has shown incredible importance in attenuating ROS-induced neurotoxicity [8, 30]. When examining GSH in our neuronal cell culture model, we observed no significant changes in any treatment group (Figure 5(a)). In contrast, when measuring GSSG we observed that PQ alone increased GSSG roughly 4-fold compared to control neurons. Moreover, low-dose AB attenuated the PQ-elevated GSSG levels back to control levels, while high-dose AB had no significant change on GSSG levels in PQ-treated cells (Figure 5(b)). Overall, these findings support our O_2_^•−^ and H_2_O_2_ data (Figures 2 and 3) and together strongly suggest that low-dose AB decreases levels of ROS, attenuates oxidative stress, and inhibits neurotoxicity following PQ exposure.

## 4. Discussion

Of the neurodegenerative diseases, Parkinson's disease is highly associated with oxidative stress induced by environmental factors such as herbicide (i.e., PQ) exposure [31]. While the exact cause of Parkinson's disease remains elusive, numerous studies have elucidated excess ROS production to be a potential mechanism in the loss of critical dopaminergic neurons in the substantia nigra in the brain [32]. A primary source of intraneuronal ROS, more specifically O_2_^•−^, implicated to be involved in the disease is complex I of mitochondria [33]. Complex I inhibitors (which are also found in pesticides and herbicides) such as rotenone and 1-methyl-4-phenyl-1,2,3,6-tetrahydropyridine (MPTP) create a backup of electrons in the mitochondrial respiratory chain, which further leak onto molecular oxygen generating O_2_^•−^ and induce oxidative stress [34]. Interestingly, PQ possesses a similar structure to MPTP and has also been demonstrated to interact with complex I to generate reactive radical species [35]. Herein, we confirm these findings by demonstrating that mitochondrial O_2_^•−^ is indeed increased in NG108-15 cells treated with PQ. Intriguingly, we observed no change in mitochondrial H_2_O_2_ levels, which suggests a predominantly 1 electron transfer to generate primarily O_2_^•−^. Moreover, low doses of AB were able to significantly attenuate this increase in mitochondrial oxidative stress, which translated to a more reducing cellular environment as evidenced by lower DHE oxidation as well as decreased levels of oxidized glutathione. In contrast, high doses of AB could not rescue the PQ-induced oxidative stress and exacerbated some of the effects. These findings warrant examination into the specific components of the AB concentrate to elucidate potential molecules that could exacerbate redox cycling reactions in a dose-dependent manner.

There are currently limited medical therapies for the treatment of neurodegenerative diseases. On the contrary, a breadth of evidence exists suggesting dietary intake of polyphenols may have beneficial effects in counteracting neurological disorders. For example, consumption of red wine, which is known to possess high levels of polyphenols, may reduce the incidence of neurological disorders [36, 37]. Other studies have demonstrated intake of polyphenol-rich foods may preserve cognitive function, delay the onset, or even reduce the risk of neurodegenerative diseases like age-related dementia or Alzheimer's disease [38–40]. However, it remains controversial if the beneficial effects of polyphenol-rich diets are actually acting in the brain, as it is not clear if polyphenols cross the blood brain barrier [41]. Polyphenols have been reported to be poorly absorbed by the intestines, rapidly excreted, and exist in low concentrations in systemic circulation [42, 43], which further argues for a potential limited role in the brain. In contrast, several investigations have concluded that low concentrations of polyphenols do in fact cross the blood brain barrier under both experimental* in situ* conditions and after* in vivo* dietary consumption of polyphenol-rich foods [44–47]. In the present study, we identified that only low concentrations of AB provided a protective role against ROS-induced neuron cell death caused by PQ. With the understanding that only small amounts of polyphenols may reach the brain after dietary consumption of polyphenol-rich foods, our data support a beneficial and antioxidant effect of these molecules in low concentrations and the possible protection against neuron cell death.

The use of antioxidants as therapeutics is controversial due to an extensive list of failed clinical trials in an array of diseases. Based on this, it is easy to conclude that antioxidants are not sufficient in ameliorating disease, but numerous variables must be taken into account when assessing the efficacy of these trials. The first variable to consider is dosage. It is commonly presumed in medicine that if a positive dose response to a drug is achieved at low concentrations then high concentrations will produce an even more favorable outcome, but this is not always found to be true. For example, in 2002 a phase II, double blind, randomized, and placebo controlled clinical trial was performed on the potential effectiveness of coenzyme Q_10_ in slowing the progression of Parkinson's disease [48]. A negative correlation was observed with increasing dose of coenzyme Q_10_ (ranging from 300 to 1200 mg/day) and progression of the disease, which thus prompted researchers to investigate even higher doses of coenzyme Q_10_ in Parkinson's disease. In 2007, another phase II, double blind, randomized, and placebo controlled study was performed utilizing doses of coenzyme Q_10_ ranging from 2400 to 4000 mg/day and found no significant improvement with any dose on the diminution of progression of Parkinson's disease [49]. The conclusion drawn from this study was that coenzyme Q_10_ provided no benefit over placebo in Parkinson's disease due to the fact that high doses could not replicate what was seen in the lower dose clinical trial. Another example of dosage discrepancies involves the use of vitamin E for therapy in Alzheimer's or Parkinson's disease patients. Three separate clinical trials utilizing vitamin E supplements (ranging from 800 to 2000 IU/day) found no significant impact or even worsening of the severity of Alzheimer's or Parkinson's disease progression [50–52]. However, three separate studies utilizing vitamin E administration through means of dietary intake (ranging from 5 to 15 mg/day in foods naturally containing higher levels of vitamin E) showed positive benefits in slowing the progression of both diseases [53–55]. Similar to what was observed with coenzyme Q_10_, it appears that lower doses (and possibly vehicle of administration) are possibly more efficacious than higher doses when examining the effects of antioxidants. In our study presented here, we observe a similar phenomenon where only low-dose AB ameliorated PQ-induced neurotoxicity, while higher doses exacerbated the phenotype. This nonlinear regression between antioxidant dosage and disease outcome may explain the subjective failure of antioxidant clinical trials and warrants further investigation into the potential mechanisms leading to the nonmonotonic response.

Another significant variable in the outcome of antioxidant therapy is the timing of administration. The majority of clinical trials focus on the treatment of patients that have already been diagnosed with a major disease, and as such assessing the preventative capabilities of antioxidants is already past due. Conversely, numerous retrospective analyses have examined the potential for dietary intake of antioxidants in altering the risk of developing neurodegenerative disorders like Alzheimer's disease. For example, it has been shown that diets rich in fruits and vegetables reduce cognitive decline and the risk for Alzheimer's disease later in life [56, 57]. Additionally, in the aforementioned Rotterdam study it was observed that intake of vitamin E in the form of food (not supplements) also reduced the incidence of dementia [54]. These studies suggest that antioxidants serve as preventative measures as opposed to reactive measures against neurological disorders. Herein, we present evidence that supports this hypothesis as we show pretreatment of neurons with AB for 24 hours prior to PQ administration protects neurons from ROS-induced cell death. Performing the converse experiment in which AB was administered at the same time or 24 hours after PQ treatment did not produce any observable beneficial response (data not shown). Taken together, antioxidant supplementation through dietary intake appears to play a greater role in the prevention of neurological diseases as opposed to their treatment.

The last major variable to consider when assessing the efficacy of antioxidants in the treatment of diseases is the specific ROS that is being targeted. ROS are often considered a homogenous group of substances that are harmful to the cell, but this view overlooks the vast complexity of the redox environment. ROS are diverse with some being free radicals, possessing charges, or participating in one or two electron oxidation/reduction reactions depending on the structure of the specific species [58]. Additionally, not all ROS cause “oxidative stress,” which is defined as irreversible damage to cellular components, but many ROS participate in controlled, regulated, and reversible modifications to cellular constituents that lead to redox-mediated signaling pathways [59]. For example, H_2_O_2_ oxidizes reduced cysteines in proteins creating reversible adducts that may alter the shape and function of a protein, thus making the protein redox responsive [60]. In contrast, O_2_^•−^ is a poor oxidant but reacts readily with iron-sulfur cluster containing enzymes reversibly affecting their activity and contributing to redox-mediated cellular signaling [61]. With the understanding that ROS-mediated reactions are unique and diverse, it becomes clear that the use of a generalized antioxidant that may scavenge several ROS at once (or potentially a ROS that is not highly relevant in the disease state) may not prove to be efficacious or even deleterious. In our data set, we demonstrate that the primary ROS produced by PQ is O_2_^•−^, and this has been shown by others as well [26]. Low doses of AB demonstrated the ability to significantly attenuate PQ-induced O_2_^•−^ in neurons, yet, high doses potentiated the production. Moreover, high dose of AB appeared to significantly reduce the amount of steady-state H_2_O_2_ in neurons even in the absence of PQ suggesting that high dosage of antioxidants altered normal redox signaling within the cells or even created a reductive stress upon the cells [62]. In summary, it appears that low, but not high, dose of AB restores the homeostatic redox environment and decreases cellular death caused by the PQ-induced O_2_^•−^-mediated oxidative stress.

Next, we observed an interesting phenomenon that Nox2 protein was virtually absent in neurons treated with high doses of AB (independent of PQ treatment). Polyphenols have been demonstrated to attenuate Nox activity in various models, but their role in regulating actual protein levels is unclear [63–65]. Our data suggest that AB may be interfering with the normal expression of Nox2, but it is unclear at this time if this occurs at the transcriptional, posttranscriptional, translational, or posttranslational level. Furthermore, the Nox2 catalytic subunit of the Nox complex is also known as gp91phox due to the fact that the 55 kDa protein becomes heavily glycosylated causing it to run on a western blot at approximately 91 kDa [66]. Polyphenols have been shown to interfere with and reduce the amount of advanced end glycation products observed in several disease states [67–69], which raises the question if these small molecules also play a role in modifying normal cellular glycosylation of proteins. Our data suggest AB plays a significant role in the downregulation of Nox2, and further investigation is warranted into the mechanism of this process.

Finally, our study does possess some potential limitations. First, due to proprietary reasons we are limited in the understanding of the exact constituents and concentrations of the commercially available AB concentrate. Additionally, while the dilutions we utilized did produce favorable outcomes, further biodistribution studies are needed to understand if the optimal concentrations we observed translate* in vivo*. Next, our use of a neuronal cell line may not perfectly mimic the effects on primary neurons. However, NG108-15 cells divide and grow in a highly differentiated manner, which increases their likelihood to react like primary neurons in an* in vitro* setting. Lastly, treatment of neuronal cells* in vitro* with AB does not take into account* in vivo* variables such as absorption and biotransformation that may alter the AB components and exposure to neurons in a living system. Upon consumption, polyphenols may be oxidized by liver enzymes and the digestive microbiota, which could ultimately change the structure and function of these molecules once they have reached a target organ. While our current studies do not address the potential alterations digestion may have on the AB, we believe the data presented herein show significant preliminary promise for AB in the amelioration of ROS-induced neurotoxicity. With these promising results, we are currently investigating the ability of AB to attenuate neurological dysfunction* in vivo* utilizing various animal models of neurodegeneration. These models will allow for a deeper understanding regarding AB bioavailability to neurons of the central nervous system, and if concentrations are able to reach levels necessary for the attenuation of oxidative stress-mediated neurological disease.

## Abbreviations

PQ: Paraquat
O_2_^•−^: Superoxide
ROS: Reactive oxygen species
AB: Aronia berry concentrate
DHE: Dihydroethidium
H_2_O_2_: Hydrogen peroxide
CuZnSOD: Copper/zinc superoxide dismutase
MnSOD: Manganese superoxide dismutase
GSH: Reduced glutathione
GSSG: Oxidized glutathione
Nox: NADPH oxidase.
